# Reproductive performance of single fixed-time inseminated sows with semen doses submitted to different semen storage times

**DOI:** 10.1590/1984-3143-AR2022-0059

**Published:** 2023-01-13

**Authors:** Matheus Schardong Lucca, Rafael Dal Forno Gianluppi, Ana Paula Gonçalves Mellagi, Fernando Pandolfo Bortolozzo, Ivo Wentz, Rafael da Rosa Ulguim

**Affiliations:** 1 Setor de Suínos, Departamento de Medicina Animal, Faculdade de Veterinária, Universidade Federal do Rio Grande do Sul, Porto Alegre, RS, Brasil

**Keywords:** extender type, storage time, total born

## Abstract

This study aimed to evaluate the reproductive performance of sows submitted to single fixed-time insemination (SFTAI) using boars according to capacity for liquid *in vitro* semen preservation, type of extender, and storage time. Boars (n = 12) were classified into two groups based on progressive motility (PM) at 120 h of semen storage: low (PM - 64.5%) - and high-preservation (PM - 83.9%) capacity for semen storage. Weaned sows (n = 397, parity - 1 to 7) were inseminated (1.5×10^9^ sperm cells) in a factorial design: two classes of boars (low- or high-preservation), two types of extenders (short- or long-term), and two semen storage times at insemination (24 or 72 h). An adapted triptorelin acetate protocol was used for SFTAI. Total sperm motility (TM) and PM at insemination were greater in high-preservation boars at 72 h compared with low-preservation boars at 24 or 72 h (P < 0.01). Short- or long-term extender did not affect (P ≥ 0.68) TM and PM in high-preservation boars; however, long-term extender improved these parameters in low-preservation boars (P < 0.01). Pregnancy and farrowing rates were not affected by groups (P > 0.05). Total piglets born (TPB) was reduced (P = 0.05) in low-preservation boars with 72 h of storage (13.6 ± 0.5) compared to high-preservation boars with semen stored for 24 or 72 h (15.2 ± 0.5 and 15.5 ± 0.5, respectively). The low-preservation boars reduced the TPB in sows submitted to SFTAI, and this reduction was greater using semen stored for 72 h.

## Introduction

Reduced boar semen quality is associated with poor reproductive performance which could be related to individual characteristics (Roca et al., 2016) and/or semen processing and storage (Yeste et al., 2017). Differences in the preservation of boars to liquid semen storage also impact reproductive performance (Ruiz-Sánchez et al., 2006). According to Lucca et al. (2021), boars with high-preservation to semen storage had more total piglets born (TPB) than low-preservation boars. The type of extender could mitigate the negative impacts of semen storage. Long-term extender improved total sperm motility in boars with low-preservation to semen storage (Lucca et al., 2021). Long-term extender is also associated with fertility improvement (Pinart et al., 2015). Detrimental impacts on reproductive performance using multiple inseminations with semen doses stored beyond 72 h have not been observed (Anil et al., 2004; Lucca et al., 2020). However, these studies did not explore the differences among boars regarding capacity for semen preservation to storage and use of single fixed-time insemination (SFTAI) with different types of extenders and storage times.

SFTAI in swine is a promising technology and studies for improving the reproductive performance of this technique are necessary. Recommendations regarding storage time of the semen doses used for SFTAI are scarce in the literature. Therefore, this study aimed to evaluate the reproductive performance of SFTAI using boars classified as low- or high- preservation to liquid semen storage with the ejaculates being diluted in short- or long-term extenders and stored for 24 or 72 h.

## Methods

This project was approved by the Federal University of Rio Grande do Sul’s Ethics Committee on the Use of Animals (34329).

### Animals and farms

Boars (AG 337 - Agroceres PIC^®^) were individually housed in stalls (2.4 × 0.7 m) in a temperature-controlled barn (18-23 °C). They were fed twice a day with a corn-soybean based diet (12.5% CP, 0.68% SID Lysine and 3.25 Mcal ME/kg) and *ad libitum* access to water.

A total of 397 crossbred weaned sows (Camborough - Agroceres PIC^®^) were used for SFTAI during six weeks (spring season). After weaning, the sows were housed in individual crates (2.2 × 0.6m) for estrus detection, breeding, and gestation. They were fed with automatic feeders with a corn-soybean diet (14% CP, 0.62% SID Lysine and 3.30 Mcal ME/kg) and *ad libitum* access to water.

### Experimental design

Twelve boars, from a population of 32 boars, were classified based on progressive sperm motility (PM) at 120 h of storage using Beltsville Thawing Solution (BTS) in two classes: low- (mean PM 64.5%, range 54.9-70.6%) and high-preservation boars (mean PM 83.9%, range 77.5-88.9%). The low- and high-preservation groups were classified based on a ranking of the average of PM at 120 h of storage (top and bottom six boars) in three semen collections per boar performed once a week (Lucca et al., 2021). All semen analyses were performed using the CASA System (Sperm Vision^®^ 3.7; MOFA Global, Verona, WI, USA) using settings reported by Lucca et al. (2021). After classification, six ejaculates of each class (collection once a week) were diluted on a split-sample basis using a short-term (BTS) or a long-term extender (Androstar^®^ Plus, Minitub GmbH, Tiefenbach, Germany). The semen doses were produced with 1.5 × 10^9^ total sperm cells (50 ml) and stored at 17 ± 1 °C. Total sperm motility (TM) and PM were assessed at 24 and 72 h of storage using the CASA System (Sperm Vision^®^ 3.7; MOFA Global, Verona, WI, USA).

The weaned sows were randomly assigned in a factorial design (2 × 2 × 2): two classes of boars (low- or high-preservation), two types of extenders (short- or long-term), and two storage times (24 or 72 h). Sows were distributed among groups (~50/group) according to parity (1 to 7), lactation length (18 to 23 days), and previous total number of piglets born (≥ 9). Detection of estrus started on the weaning day and was performed once a day (0800 h) using fence-line boar contact and the back-pressure test. Multiparous weaned sows were submitted to the following SFTAI protocol: sows in estrus within 72 h post-weaning were excluded; the remaining sows were treated with 200 µg of intravaginal triptorelin acetate (OvuGel^®^, JBS United Animal Health, Sheridan, IN, USA) at 96 h post-weaning (Knox et al., 2011); sows in estrus 22-24 h after the triptorelin acetate application were SFTAI in each group. Semen doses from the same ejaculate stored at 24 or 72 h were used trough intrauterine insemination. Pregnancy rate (PR), farrowing rate (FR), and TPB were recorded. Pregnancy rate was performed by the same trained technician using transcutaneous real-time ultrasound and convex linear transducer (A6V, SonoScape^®^ Co. Ltda, Shenzhen, China) at 25 days post-insemination.

### Statistical analysis

The Statistical Analysis System software (SAS^®^, version 9.4) using the GLIMMIX procedure was performed in all analysis. Data are expressed as mean ± standard error (SE). Class of boar, type of extender, storage time, and their interactions were considered as fixed effects and the week as a random effect in the model. Total motility and PM were analyzed in a binomial distribution; PR and FR by logistic regression using a binary distribution. The TPB was analyzed in a normal distribution and means compared by the Tukey-Kramer test. Differences were statistically significant at the 95% confidence level (P ≤ 0.05). The statistical model has considered the 3-way interaction and the results explored according to the main significant effect (3- or 2-way interaction).

## Results

Parity (3.2±1.5), lactation length (20.2±1.1), and previous TPB (15.6±2.6) (P ≥ 0.28) in the sows used did not differ among groups. The TM and PM were not affected (P ≥ 0.68) by class of boar, type of extender, storage time in the 3-way interaction. However, semen doses from high-preservation boars at 24 h had higher (P < 0.01) TM and PM (94.4 ± 0.5%; 87.4 ± 1.1%, respectively) compared to high-preservation boars at 72 h (91.5 ± 0.8%; 83.4 ± 1.3%, respectively; Table 1). High-preservation boars at 24 h also had higher TM and PM than low-preservation boars at 24 h (88.2 ± 1.0%; 75.7 ± 1.7%, respectively) or 72 h (85.7 ± 1.3%; 72.8 ± 1.9%, respectively). The use of short- or long-term extender did not affect TM (93.1± 0.7%; 93.1± 0.7%, respectively) or PM at insemination (85.7±1.2%; 85.4±1.2%, respectively) in the high-preservation boars; however, in the low-preservation boars the long-term extender improved TM (87.8± 1.1%) and PM (75.7±1.8%) compared to the use of short-term extender (86.2± 1.1%; 72.8±1.8%; respectively). In addition, the semen doses from high-preservation boar at 24 h have 94.4±0.5% and 87.4±1.0%, for TM and PM respectively, higher than semen doses from high-preservation boars at 72h of storage and low-preservation boar at 24 h and 72 h (Table 1). The long-term extender at 24 h of storage have the higher TM and PM compared to short-term extender at 24 h and the long or short-term extender at 72 h.

**Table 1:** Sperm motility and reproductive performance of weaned sows single inseminated in 2-way interaction: class of boar (low- or high-preservation to semen storage), type of extender (short- or long-term), and time of semen storage (24 or 72 h).

**Variables**	**Low-preservation**		**High-preservation**		**Low-preservation**		**High-preservation**		**Short-term**		**Long-term**		**P < 0.05**
	**ST**	**LT**	**ST**	**LT**	**24**	**72**	**24**	**72**	**24**	**72**	**24**	**72**	
n	97	99	97	95	94	102	90	102	91	101	91	103	-
TM, %	86.2±1.2^c^	87.8±1.1^b^	93.1±0.7^a^	93.1±0.7^a^	88.2±1.0^c^	85.7±1.3^d^	94.4±0.5^a^	91.5±0.8^b^	91.3±0.8^b^	89.0±1.0^c^	92.4±0.7^a^	89.0±1.2^c^	C×E; C×T; E×T
PM, %	72.8±1.8^c^	75.6±1.8^b^	85.3±1.2^a^	85.7±1.2^a^	75.7±1.7^c^	72.8±1.9^d^	87.4±1.0^a^	83.4±1.3^b^	81.0±1.4^b^	78.9±1.5^c^	83.4±1.3^a^	78.2±1.6^c^	C×E; C×T; E×T
PR, %	95.4±2.4	95.0±2.2	94.3±2.3	87.3±3.3	93.2±2.7	96.6±1.9	90.0±3.0	92.4±2.8	90.7±3.0	97.2±1.6	92.7±2.8	90.8±3.0	-
FR, %	93.6±2.5	91.7±2.7	91.4±2.8	85.0±3.5	90.4±3.0	94.4±2.3	86.9±3.3	90.1±3.1	88.7±3.2	95.2±2.1	88.9±3.2	88.7±3.2	-
TPB	14.3±0.5	14.2±0.5	15.4±0.5	15.4±0.5	14.8±0.5^ab^	13.6±0.5^b^	15.2±0.5^a^	15.5±0.5^a^	15.4±0.5	14.2±0.5	14.6±0.5	14.9±0.5	C×T

Values expressed as LSMeans ± SE; Class of boar (C) defined according to progressive sperm motility at 120 h of storage using Beltsvile Thawing Solution extender (six boars in each class); Type of extender (E) has considered the use of short- (ST) or long-term (LT) extender (BTS or Androstar Plus, respectively); Storage time (T) of semen doses at the moment of insemination. TM: total motility at insemination; PM: progressive motility at insemination; PR: pregnancy rate; FR: farrowing rate; TPB: total piglets born. There is no effect for 3-way interaction (P ≥ 0.15). a-c: Differs significantly in the line (P ≥ 0.05).

**Figure 1:**
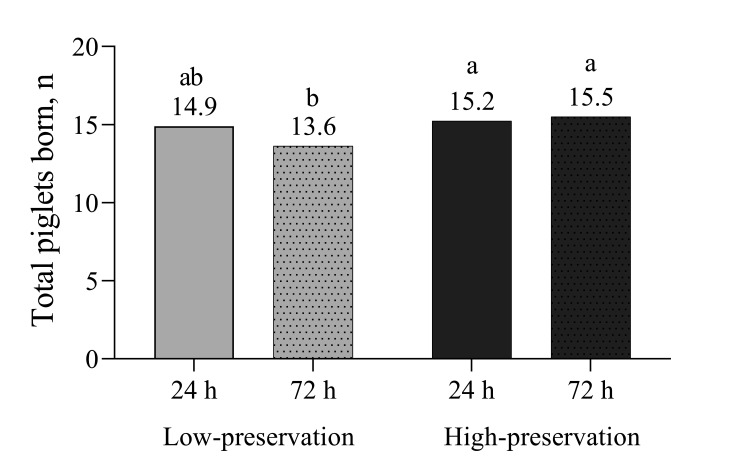
Total piglets born from boars classified as low- or high-preservation of the semen doses and the use of semen doses with 24 h or 72 h of storage for a single fixed-time insemination in weaned sows (n = 397). ab: Differs significantly (P = 0.05).

The PR and FR were not affected by class of boar, type of extender, storage time or their interaction (Table 1). However, sows inseminated with semen doses from the low-preservation boars with 72 h of storage had fewer TPB compared to high-preservation boars stored for 24 or 72 h (P = 0.05). Semen from low-preservation boars at 24 h compared to 72 h of storage did not differ regarding TPB (Figure 1).

## Discussion

Differences in sperm motility among the evaluated factors were observed, but all values were superior to the recommendation to avoid impairments in reproductive performance (Flowers, 1997). Even though considering this response, the results indicated lower reproductive performance when SFTAI was used with semen doses from low-preservation boars stored for 72 h. The use of a long-term extender resulted in a slight increase in TM and PM at insemination for the low-preservation boars but did not improve the reproductive performance. These results corroborate other studies (Pinart et al., 2015; Lucca et al., 2021) in which the sperm parameters were improved without affecting the reproductive performance when long-term extender was used. In this study, the main effect on reproductive performance was associated with the class of boars and semen storage time.

The aging of semen doses has been related to decreased reproductive performance (Waberski et al., 1994; Anil et al., 2004). The fertility was reduced when semen doses extended in BTS were used beyond 48 h of storage in gilts single inseminated immediately after ovulation (Waberski et al., 1994). Regardless of the class of boar and extender, single inseminated sows with semen doses stored for 24 or 72 h did not affect PR and FR. However, a reduction of 1.6 piglets born was observed when semen doses from low-preservation boars were used at 72 h of storage compared to high-preservation boars (24 or 72h). According to Haugan et al. (2005), the FR was not affected but the TPB was reduced when semen doses with BTS were used beyond 48 h using single insemination. In long-term extender (Androhep), the effect on fertility compared to BTS was evident only when the semen doses were used with more than 96 h of storage (Haugan et al., 2005). Different from the previous study, we classified the boars based on the capacity of preservation to semen storage. According to Lucca et al. (2021), boars with low capacity for maintaining sperm motility during storage also had an impairment on TPB even using only sows in estrus in the STFAI protocol to minimize the chance of an insemination out of the optimal interval in relation to ovulation. In addition, we cannot disregard a possible individual effect of boars presenting reciprocal chromosomal translocation and the association with reduced TPB (Rodríguez et al., 2010), especially in the low-preservation class of boars. Thus, it was not possible to explain the reasons for reduced TPB in low-preservation boars in the present study. However, in a practical condition a selection of boars before the adoption of SFTAI protocols could consider the classification of boars based on PM during storage to mitigate the risk of reduced TPB.

## Conclusion

The low-preservation boars had reduced total piglets born in sows submitted to SFTAI and the reduction was higher when semen stored beyond 72 h was used.
